# Relation between Density and Compressive Strength of Foamed Concrete

**DOI:** 10.3390/ma14112967

**Published:** 2021-05-31

**Authors:** Rokiah Othman, Ramadhansyah Putra Jaya, Khairunisa Muthusamy, MohdArif Sulaiman, Youventharan Duraisamy, Mohd Mustafa Al Bakri Abdullah, Anna Przybył, Wojciech Sochacki, Tomasz Skrzypczak, Petrica Vizureanu, Andrei Victor Sandu

**Affiliations:** 1 Faculty of Civil Engineering Technology, Universiti Malaysia Pahang, Kuantan 26300, Pahang, Malaysia; khairunisa@ump.edu.my (K.M.); mdarif@ump.edu.my (M.S.); 2 Department of Civil Engineering, College of Engineering, Universiti Malaysia Pahang, Kuantan 26300, Pahang, Malaysia; youventharan@ump.edu.my; 3 Center of Excellence Geopolymer and Green Technology (CEGeoGTech), Universiti Malaysia Perlis (UniMAP), Kangar 01000, Perlis, Malaysia; mustafa_albakri@unimap.edu.my (M.M.A.B.A.); peviz@tuiasi.ro (P.V.); sav@tuiasi.ro (A.V.S.); 4 Faculty of Chemical Engineering and Technology, Universiti Malaysia Perlis (UniMAP), Kangar 01000, Perlis, Malaysia; 5 Department of Physics, Częstochowa University of Technology, 42-201 Częstochowa, Poland; anna.przybyl@pcz.pl; 6 Faculty of Mechanical Engineering and Computer Science, Częstochowa University of Technology, 42-201 Częstochowa, Poland; wojciech.sochacki@pcz.pl (W.S.); t.skrzypczak@imipkm.pcz.pl (T.S.); 7 Faculty of Materials Science and Engineering, Gheorghe Asachi Technical University of Iasi, 700050 Iasi, Romania

**Keywords:** relation, density, compressive strength, workability, foamed concrete, processed spent bleaching earth

## Abstract

This study aims to obtain the relationship between density and compressive strength of foamed concrete. Foamed concrete is a preferred building material due to the low density of its concrete. In foamed concrete, the compressive strength reduces with decreasing density. Generally, a denser foamed concrete produces higher compressive strength and lower volume of voids. In the present study, the tests were carried out in stages in order to investigate the effect of sand–cement ratio, water to cement ratio, foam dosage, and dilution ratio on workability, density, and compressive strength of the control foamed concrete specimen. Next, the test obtained the optimum content of processed spent bleaching earth (PSBE) as partial cement replacement in the foamed concrete. Based on the experimental results, the use of 1:1.5 cement to sand ratio for the mortar mix specified the best performance for density, workability, and 28-day compressive strength. Increasing the sand to cement ratio increased the density and compressive strength of the mortar specimen. In addition, in the production of control foamed concrete, increasing the foam dosage reduced the density and compressive strength of the control specimen. Similarly with the dilution ratio, the compressive strength of the control foamed concrete decreased with an increasing dilution ratio. The employment of PSBE significantly influenced the density and compressive strength of the foamed concrete. An increase in the percentage of PSBE reduced the density of the foamed concrete. The compressive strength of the foamed concrete that incorporated PSBE increased with increasing PSBE content up to 30% PSBE. In conclusion, the compressive strength of foamed concrete depends on its density. It was revealed that the use of 30% PSBE as a replacement for cement meets the desired density of 1600 kg/m^3^, with stability and consistency in workability, and it increases the compressive strength dramatically from 10 to 23 MPa as compared to the control specimen. Thus, it demonstrated that the positive effect of incorporation of PSBE in foamed concrete is linked to the pozzolanic effect whereby more calcium silicate hydrate (CSH) produces denser foamed concrete, which leads to higher strength, and it is less pore connected. In addition, the regression analysis shows strong correlation between density and compressive strength of the foamed concrete due to the R^2^ being closer to one. Thus, production of foamed concrete incorporating 30% PSBE might have potential for sustainable building materials.

## 1. Introduction

In recent years, the world has moved in a new direction by looking for lighter, durable, practical, economical, and environmentally sustainable materials to cater to the demands of modern construction. It is well known that concrete is a massive substance, and it is the main component used for construction. As a result, lightweight concrete has been introduced in the construction industry owing to its lower density, ease of handling, and most importantly, cost savings. Foamed concrete (FC) is a type of lightweight concrete which is produced through the combination of cement paste and preformed foams that cause the foam concrete to be lighter than normal concrete [Brandt, 2009]. The best thing about foamed concrete is that it can be placed easily by pumping, if necessary, and does not require compaction, vibration, or levelling. It can be a highly workable concrete. Due to its porous or cellular make up, it provides significant benefit to the construction industry through its unique properties of being of a low density, flowing and self-compacting [1,2], thermally superior, and having excellent sound insulation properties [3,4,5]. It is commonly used in buildings located in cold regions because it has excellent resistance against water and frost action in moist conditions because its air voids act as empty chambers in the paste for the freezing and migrating water to enter; thus, the pressure in the pores will be relieved and will prevent the concrete from damage. In addition, it can reduce the energy that is used to cool and heat the building [4].

Recently, a concrete containing pozzolanic material used as a cement replacement as a building material for the construction industry has been another approach for reducing greenhouse gases (GHG) emissions including carbon dioxide (CO_2_). The incorporation of waste or industrial by-products, such as fly ash [6,7], silica fume [8], ground granulated blast furnace slag [9], rice husk ash [10], sewage sludge ash [11], sludge paper mill [12], graphite tailing [13], palm oil fuel ash [14], and soil [15] and sand [5] replacements, in FC has been introduced. According to Richard and Ramli [16], Richard and Ramli [17], and Bayuaji [18], as compared to normal concrete, FC is considered as green concrete and economical due to its no aggregate content, and its sand and cement can be replaced by the use of recycled material. In addition, FC does not impose high loadings and the weight of the structure built is reduced due to the low density of its concrete. Therefore, when using FC, more benefits are gained because reduction to the dead load of the building will also result in reduction to the cost of materials, as well as the reinforcement steel cost, and the timing of the project [19].

In FC, the compressive strength reduces with decreasing the density [20]. Dransfield [21] and McCarthy and Jones [19], found that the strength of FC with densities ranging between 400 and 1600 kg/m^3^ is between 1 and 10 MPa, which is adequate for its purpose as bulk filling, void filling, stabilization and insulation material, bridge abutment backfill, slab and housing insulation, and other underground works. Therefore, Jones and McCarthy [19], and Shannag [22] point out that FC can be used as a structural application if the compressive strength turns out to be 25 MPa. This development process has grown globally with some advances in the specification of foamed concrete published by Jones and McCarthy [19], with details of its materials and method of production published by Brady and Greene [22,23,24,25,26], and engineering properties of foamed concrete and applications being reported by [27,28,29,30,31]. In relation to this matter, increasing cement content increased the compressive strength of foamed concrete. The trend is similar in concrete, whereby Neville [32] reported that higher cement content leads to the increase in the compressive strength of normal concrete. However, Jones [33] said that the strength increase was found to be minimal above a cement content of 500 kg/m^3^. If the amount of cement can be reduced or partially replaced with pozzolan material, a more environmental friendly FC can be produced. Therefore, this research investigates the effect of Processed Spent Bleaching Earth (PSBE) as a partial cement replacement on the workability, density, and compressive strength of FC.

PSBE is derived from waste material known as SBE from the palm oil industry, which causes environmental pollution as it is disposed at landfill. Globally, an estimated 2 million tons or more of SBE are utilized worldwide in the refining process based on worldwide production of more than 200 million tons of oils, which is equivalent to 1% mass of SBE being produced relative to the amount oil produced yearly [34]. There are 423 palm oil mills in Malaysia that leads to the production of an estimated 240,000 tons per annum or more of SBE in the refining process of crude palm oil [35]. According to Eliche-Quesada and Corpas-Iglesias [36], SBE can present a potential fire and pollution hazard because it contains 20 to 40% residual oil by weight, and metallic impurities and organic compound upon its disposal. Thus, use of PSBE as partial cement replacement in FC would be able to reduce waste ending at landfill and reduce cement usage. In addition, the main components of SBE are silica and alumina that enhance the pozzolanic reactivity, which is useful to improve the strength and durability of FC. The construction industry has its highest opportunity to reduce CO_2_ emission by delivering green technology and green living.

In general, the properties of concrete depend on the properties of its constituent materials. However, the method used to design normal concrete mix cannot be used for designing a FC mix since it contains no coarse aggregate [37]. The foamed concrete mix design is commonly determined based on a trial and error approach [38,39,40,41,42,43,44]. The design of the experiment based on empirical or computational modelling [45,46,47,48] and statistical methods approaches have been implemented to analyze multifactor experiments and the model used in the prediction of compressive strength of foamed concrete with minimal mean square errors and standard deviation. In addition, the analysis of variance (ANOVA) is used to ascertain the influence of different factors on the various properties to get the optimal conditions for the target value, and the multiple regression approach is used to develop empirical relationships that are used for the mix design [49,50,51].

According to previous studies [52,53,54,55,56,57,58,59], the designed density was set due to the particular practice of foamed concrete. For instance, in order to obtained compressive strength of 17 MPa or higher at 28 days for the structural usage, the density should be controlled in the range of 1500 to 1800 kg/m^3^. ASTM C796-19 [60] and ACI 523.3R-14 [61] stated that the mix proportioning of foamed concrete started with the set up of its plastic density, cement content, water to cement ratio based on volumetric rather than weight for density (D, kg/m^3^), cement content (C, kg/m^3^), water (W, kg/m^3^), and sand (S, kg/m^3^). While, the compressive strength can be increased based on altering the constituents materials for a given density even though the strength of foamed concrete depends on its density. Usually, the mixture design strategy of the mortar or base mix (cement, sand, or any other filler and water) defines the strength of the FC. The target density, water and sand are calculated from the Equation (1) to Equation (2) [44].

Target plastic density,

D = C + W + S(1)

where (C) represent cement content + any cement replacement (Rc), (W) water content and (S) sand content + any sand replacement (Rs).

Water content,

W = (*w/c*) × (C + Rc + Rs) (2)

where (*w/c*) represent water to cement ratio, (C) cement content, (Rc) any cement replacement and (Rs) any sand replacement.

Furthermore, curing condition is one of the factors that influence the strength of FC. Curing is defined as the process of controlling the moisture and temperature during the cement hydration. James et al. [62,63] studied the effect of different curing condition on the compressive strength of concrete. The water curing is the best curing condition for normal concrete to produce higher compressive strength, followed by wet covering and sprinkling having the least compressive strength. Several researchers [51,55,64,65] have reported several findings on curing conditions for FC such as water curing, sealed curing, air curing, moist curing, steam curing whether at atmospheric pressure or high pressure (also called autoclaving).

According to Brady et al. [24], the water curing exhibit lower strength compare with cured at 50 °C and sealed in plastic bag because of the built up of pore water pressure in the saturated microstructure of the FC. While higher strength of FC can be produced by using air curing at 50 °C and sealed in a plastic bag at a constant temperature of 22 °C. The similar trend has been reported by Falliano et al. [66] who found that air curing lead to higher compressive strength, while cellophane and water curing conditions exhibited poor compressive strength. Moreover, Kado et al. [67] reported that the air cured FC is more stable than water cured specimen for all the densities. Other researcher Hu, Li, Liu, and Wang [68] found that the specimen cured in high humidity produces denser pore and higher compressive strength for low density FC. However, the combination of water and then followed by air curing, would increase the compressive strength of FC as the ages increased and achieved the ultimate strength [55].

All the reviewed above found that the properties of foamed concrete were influenced by the mix proportion components such as sand to cement ratio, water to cement ratio, foam volume, and binder and filler content. In addition, the employment of pozzolan in foamed concrete has demonstrated significant influence in improving the workability, compressive strength, and durability due to the ability of silica in the pozzolan material to convert the CH to CSH depends on the amorphous state, the amount of silica content and specific surface area. The pozzolanic reaction improves the properties of FC through the formation of additional CSH gel. The microstructure of the hardened paste of FC became denser because the larger spaces had been filled with CSH gel, and the capillary voids decreased and reduced in size. The denser structure leads to improve the strength and durability of FC. However, the effect of PSBE as partial cement replacement on the properties of FC is not yet available. Therefore, this research attempts to fill the gaps of knowledge by studying the effect of PSBE as partial cement replacement in FC on its workability, density, and compressive strength. This study aims to obtain the relationship between density and compressive strength of foamed concrete. Finally, the incorporation of PSBE as cement replacement can promote the use of waste materials and lead to reduce the CO_2_ emissions, and conserve energy and resources.

## 2. Experimental Program

### 2.1. Materials

**Figure 1:**
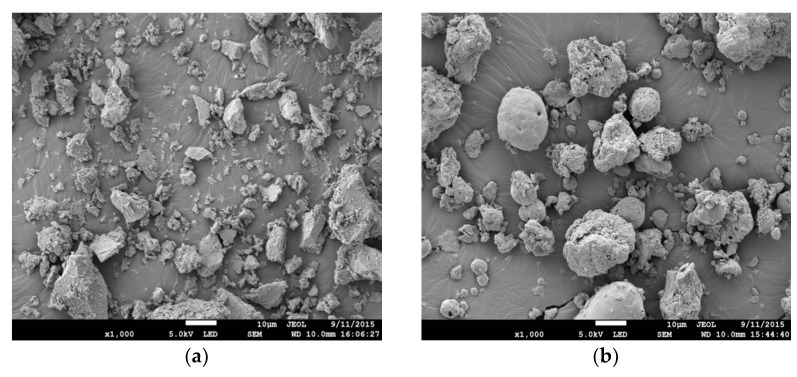
SEM micrograph of OPC and PSBE. (**a**) OPC, (**b**) PSBE.

The materials used to prepare specimens in this research are cement, water, silica sand, foaming agent, and pozzolan material known as processed spent bleaching earth (PSBE). Ordinary Portland cement (OPC) produced by YTL Cement Sdn. Bhd was used throughout the experimental work conforms to BS EN 197-1:2000 Type I. Tap water was used for mixing and curing purposes. The hydrolyzed protein foaming agent was manufactured by LCM Technology Sdn. Bhd. Kuantan is conforming to ASTM C796-19 [60] The chemical composition test and porcine detected were done to ensure that the foaming agent used was approved to ASTM C869-16 [69] and was good for safety and health. Silica sand was manufactured by Johor Silica Industries Technology Sdn. Bhd with a 425 µm sieve (No.425 ASTM) conforming to BS EN 12620,2002 [70] and PSBE was provided by Eco Innovation Sdn. Bhd. The PSBE was dried in oven for 24 h at a temperature of 105 ± 5 °C then sieved through a No.300 ASTM. PSBE was classified as Class N Pozzolan in accordance with ASTM C618-12 [71] and conformed to BS Specification for Pulverized-Fuel as for use with Portland cement (BS 3892-1/BS EN 450). Table 1 shows the chemical composition and physical properties of Processed Spent Bleaching Earth. The particle distribution of PSBE was shown in Figure 1. It revealed that the particles shape of PSBE was spherical, smooth surface, and of a porous structure as shown in Figure 1b. Meanwhile, the shape of the particles for OPC consists of an angular and irregular shape as shown in Figure 1a.

### 2.2. Mix Design

In this study, the trial mix was proportioned by volume due to ACI 523.3R-14 [61] with single variable for single factor test as shown in Table 2. The control FC mix which contained only cement, sand, water, and foam was set as a reference for further study as compared with PSBE as partial cement replacement. Firstly, the different mortar mixes were prepared to obtain the optimal sand to cement ratios (*s/c*) ranging from 0.5 to 2.0, with an interval of 0.5. Table 2 shows the mix proportion for 1 m^3^. According to Kavitha and Mallikarjunrao [72], several studies have reported that, in general, the optimum water to cement ratio (*w/c*) for mortar or paste lies between 0.5 and 0.6, but with superplasticizer the *w/c* ratio lies between 0.17 and 0.19. In the mix design recommended by ACI 523.3R-14 [61], the *s/c* ratio ranged from 0.29 to 3.66 with densities ranges from 800 to 1920 kg/m^3^. Further, the cement content for the common strength FC with a density range of 1100 to 1500 kg/m^3^ were adopted at 920 to 1260 kg/m^3^. For this reason, this study chose the *s/c* ratio from 0.5 to 2.0 and *w/c* ratio at 0.5 to produce a stable mix and achieve the design density and strength. Then, the selected mortar mix was used to produce a control FC, which was based on the results of density, workability, and average 28-day compressive strength.

Secondly, the control FC mix was prepared with different percentages of foam dosage to determine the best portion of foam used in the ranges of 20 to 35% with an interval of 5 from the mortar volume. As reported by Zhao et al. [43], the wide range of densities foamed concrete (400 to 1600 kg/m^3^) were produced by adjusting the dosage of pre-formed foam added into mortar paste. In general, the stability and consistency of FC were affected by foam volume and quality of foam, which was also affected by its density. Hence, it is important that the foam stability is maintained. According to ASTM C796 [60], the density of foam ranges from 32 to 64 kg/m^3^. From the previous study, the quality of the foam is affected by its density, dilution ratio, and mixing process with the mortar. The foam should be stable and not collapse during processing and placement. However, most of the studies on FC are concentrated on the effect of admixtures on its strength and other properties of FC but seldom on the effect of foam dosage [65]. Therefore, in this study the trial mixes were carried out to determine the proper dosage of foam with foam density ranges from 50 to 60 kg/m^3^ where the maximum average can be 55 kg/m^3^. According to Maldonado-valderrama, Martı, Martı, and Cabrerizo-vı [65] and Panesar [73] the density of foam produced with protein based foaming agent is often 50 kg/m^3^ compared to synthetics. Based on previous study, the compressive strength drops sharply with an increased in foam volume ranging from 20% to 80%. It can be seen that the compressive strength of FC is influenced by foam volume. For FC with the foam volume ranging from 20% to 50%, it can reach a density of 65% to 35% of mortar and its strength within 30 to 10 MPa [8]. For this reason, this trial mix was chosen with foam dosage ranging from 20% to 35% to produce a stable mix and achieving the design density and high strength with the *w/c* ratio and dilution ratio used being 0.5 and 1:33, respectively, as recommended by the manufacturer of foaming agent. Then, the best percentage of the foam dosage was selected to produce the control FC mix, which was based on the results of density, workability, and average 28-day compressive strength.

Thirdly, a trial mix was designed to determine the optimal water to cement ratio (*w/c*) within the range of 0.4 to 0.6 with an interval of 0.05. The *s/c* ratio, dilution ratio, and percentage of foam dosage in this trial mix were kept constant throughout at 1.5, 1:33, and 25%, respectively, based on previous trial mix recommendations. Kearsley [74], Ramamurthy et al. [27] and Nambiar and Ramamurthy [75] reported that usage of too little water will lead to disintegration and too much water will lead to segregation. The water–cement ratio of the mixture controls the workability of foamed concrete. Nevertheless, the adequate workability depends upon the type of binders, desired strength required, and whether a water reducing or plasticizing agent has used. In general, the range of water–cement ratio is between 0.4 and 0.8. However, a higher value of water–cement ratio is required if finer grained binders are used, such as fly ash and slag. An increase in the water content will increase the workability of the mixture by coating the particles more thoroughly and improving the flow of the concrete. Therefore, the optimal *w/c* ratio that the control FC was studied to produce is based on the results of density, workability, and average 28-day compressive strength.

Next, a trial mix was designed to determine the optimal dilution ratio of a foaming agent in the range of 1:20 to 1:40 with an interval of 5. The sand cement ratio, water–cement ratio, and percentage of foam dosage in this trial mix were kept constant throughout at 1.5, 0.5, and 25%, respectively, based on previous trial mix recommendations. The foaming agent’s dilution ratio has a significant impact on the properties of foam, which in turn affect the fluidity, compressive strength, flexural strength, and shrinkage on the foamed concrete. According to Yu et.al [76], when the dilution ratio is increased, the fluidity of the FC slurry increases gradually; when the dilution ratio of the foaming agent is in the range of 20 to 40 and 60 to 80, it increases rapidly; and when the dilution ratio of the foaming agent varies from 40 to 60. Brady et al. [24] reported that the dilution is one part of a foaming agent to between 5 to 40 parts of water, and the range of foam density is between 20 and 90 kg/m^3^. In addition, the range of foam density from 30 to 50 kg/m^3^ and the water–cement ratio range between 0.3 and 0.5 is produced by a various surfactants [77]. In this study, protein based foaming agent at five different dilution ratios was used to produce stable foam and it did not collapse during process and placement. The foam stability produced by the protein-based foaming agent at five different dilution ratios was controlled by maintaining its foam density in the range of 50 to 60 kg/m^3^ where the maximum average was 55 kg/m^3^ in this study. The optimal dilution ratio was selected to produce the control FC was based on the results of density, workability, and average 28-day compressive strength.

Finally, the trial mix continued to obtain the best mix proportions of FC containing PSBE (PFC). Based on the previous trial mix, the *w/c* ratio, *s/c* ratio, dilution ratio, and percentage of foam dosage in this study were kept constant throughout at 0.5, 1.5, 1:25, and 25%, respectively. According to Kareem and Hilal [78,79,80], in combination with Portland cement, class C fly ash can be used as a cement replacement, ranging from 20% to 35% of the mass of cement, while class F fly ash ranging from 20% to 30% mitigates the effects of alkali silica reaction. Similar results were reported for GGBS by Awang and Aljoumaily [80] who found that cement replacement with GGBS and GBS (unground) ranged from 30% to 70% of the weight of cement exhibited, decreasing in compressive strength when the replacement level was above 30%. In comparison, the GGBS foamed concrete mixes exhibited higher compressive strength than GBS mixes at the same replacement level due to the fineness increases in the pozzolanic activity. In this study, 6 trial mixes including control mix (0% of PSBE), and 5 mixes containing PSBE replacing cement at levels of 10, 20, 30, 40, and 50 % by weight of cement were used. The properties of FC containing different percentages of PSBE (PFC1 to PFC5) were compared to control mixture M (100% OPC without foam) and FC (100% OPC with foam).

### 2.3. Sample Preparation

**Figure 2:**
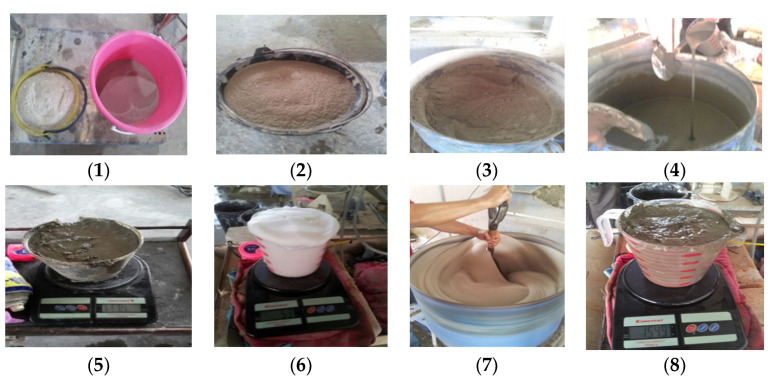
Production of foamed concrete.

The preparation of the specimens is divided into the mixing process, casting of specimens, and curing condition were reported as follows. The mixing process is presented in Figure 2. The first part is the preparation of mortar or cement paste. The mixer drum is filled with cement, silica sand, and PSBE and the constituents were dry mixed for a few minutes. Then, the water is added and mixed until the slurry becomes homogenous. The density and the workability of the slurry are measured before and after the pre-foamed foam is added. In this study, density is obtained by measuring 1 L of slurry by beaker and weighing it. The second step is the preparation of preformed foam where 1 L of a foaming agent is mixed with 25 L of water in the foam machine where the density of foam should be in the range of 50 kg/m^3^. The next stage combines the foam in the cement slurry after the flow table test has been tested. Foam is added into the cement slurry and mixed continuously until the foam is homogeneously mixed with the slurry. After that, the fresh FC density was recorded by measuring 1 L of the mix and weighing it until 1600 kg/m^3^ is achieved. The fresh mix is poured into the cube specimen size 100 × 100 × 100 mm. Then, the specimens were removed from the mold after 24 h. All the equipment, materials, and procedures in producing foamed concrete have been implemented were in accordance with ASTM C796.

## 3. Experimental Work

### 3.1. Flow Table Test

The present study was conducted to investigate the fresh properties of foamed concrete in terms of workability, consistency, and stability in the performance of flow behaviour derived from the mix design compositions. These tests were conducted to assess whether the calculated and actual water–cement ratios and foam required are sufficient. The workability of the FC was measured by using the flow table test. The flow table test consists of a flow table with a diameter of 255 mm and flows molds with a diameter of 100 mm. The flow table test was carried out by following the procedure of ASTM C1437-15 [81].

### 3.2. Density

In this study, density of FC was measured in three ways: fresh density, hardened density (air density), and oven dry density (dry density). The present investigation has followed the procedure of ASTM C796-19 [60] for determination of fresh density and ASTM C513-11 [82] for air and dry density. The fresh density is measured by filling and weighing a one-liter container with freshly mixed FC. The specimen was placed on the weight scale. The mass and dimensions of the air dry specimens were measured and recorded. Dry density was done after the air dry mass had been measured, with the specimen placed in the oven at 110 ± 5 °C for 24 h. The specimen was removed from the oven and placed in the desiccators after the specimen had cooled down. The mass and dimension of the oven dry specimen were measured and recorded at 24 h intervals until the loss in mass did not exceed 1% in 24 h. The average density of the three specimens representing each of the mixture was calculated. The oven-dry density was calculated by using Equation (3).

(3) $$\rho_{ovendry}=\rho_{air}-\rho_{oven}$$

### 3.3. Compression Strength Test

In this study, the cube specimens with dimension 100 mm × 100 mm × 100 mm were prepared and tested to determine the compressive strength of FC. The present investigation followed the procedure of ASTM C513-11 [82] for determination of compressive strength of FC. All specimens were cured and tested at age 28 days. Three specimens were tested to obtain the average compressive strength and maximum load. The compression test was performed by using a 2000 kN UTM machine with a loading rate of 3 kN/s. The average compressive strength of the three specimens representing each of the strength of the mixture was calculated. The compressive strength, σ, was calculated by using Equation (4).

σ = P/A(4)

where (σ) represents compressive strength in MPa, (P) Maximum Load of specimen in N, and (A) cross sectional area of the specimen in mm^2^.

## 4. Results and Discussions

### 4.1. Effect of Sand Cement Ratio

#### 4.1.1. Effect of Sand Cement Ratio on Workability of Mortar

The fresh density, flow table spread, number of the drop, stability, and consistency of different mortar mixes were studied. This study aimed to obtain the desired density for mortar 2100 kg/m^3^ to set a baseline to produce a control FC mix. Table 3 shows the workability of mortar at different sand to cement ratios. A flow table test was performed to determine the consistency of the workability of fresh mixed mortar as described in ASTM C 1437-15 [81]. The flow diameter was obtained by calculating the average measurement of the diameter flow of mortar in four orthogonal directions. The flow spread value has a directly proportional relationship with *s/c* ratios. Increasing *s/c* ratios will increase the flow spread of fresh mortar. From the results, the flow spread value for *s/c* 0.5, 1.0, 1.5, and 2.0 was 190 mm, 195 mm, 210 mm, and 215 mm, respectively. The higher the flow spread value, the higher the workability of fresh mortar. The flow spread value increases when the sand to cement ratio increases. However, the number of the drop of flow table decreases for the same reason. According to the results, the *s/c* = 1.5 meets the required flow spread for type M mortar as stated in ASTM C109-16 [83]. Then, it was obvious that when the *s/c* ratios increases, the design and fresh density of mortar increases with increasing sand content. The design density of mortar for *s/c* 0.5, 1.0, 1.5, and 2.0 was 1981 kg/m^3^, 2088 kg/m^3^, 2167 kg/m^3^, and 2227 kg/m^3^, respectively. Nevertheless, the fresh density of mortar was slightly lower than design density in the range of 120 kg/m^3^. According to the literature review, this condition shows the comparable results that increasing *s/c* ratios will increase the density of mortar [40]. Moreover, there is a good relationship between the stability and consistency of mortar density. The stability of mortar mixes for all sand to cement ratios are one, and the consistency is near to one. Based on data, the *s/c* = 1.5 produces the acceptable density by 2167 kg/m^3^, which is close to desired density for mortar of 2100 kg/m^3^.

#### 4.1.2. Effect of Sand Cement Ratio on Densities and Compressive Strength

The result of dry density and 28-day compressive strength of mortar specimens are shown in Table 4. The 28-day compressive strength of three cubes specimen were calculated at four different *s/c* ratios. The 28-day compressive strength with *s/c* of 0.5, 1.0, 1.5, and 2.0 was 20 MPa, 23 MPa, 28 MPa, and 34 MPa, respectively. It is observed that the 28-day compressive strength and *s/c* ratios have a directly proportional relationship. When the sand to cement ratio increases, the compressive strength of mortar increases with the increase in the sand content. The *s/c* = 2.0 mix achieved the highest compressive strength value as compared to others. It indicates that the 28-day compressive strength at four different *s/c* ratios was found to be higher than 25 MPa. On the other hand, the dry density of mortar for *s/c* 0.5, 1.0, 1.5, and 2.0 was 1955 kg/m^3^, 2056 kg/m^3^, 2130 kg/m^3^, and 2200 kg/m^3^, respectively. According to data, the dry density of mortar was slightly higher than fresh density in ranges below 100 kg/m^3^. There is a directly proportional relationship between the dry density and compressive strength of mortar. The compressive strength of mortar increases with the increase in density.

#### 4.1.3. Relationship between Compressive Strength and Density of Mortar

**Figure 3:**
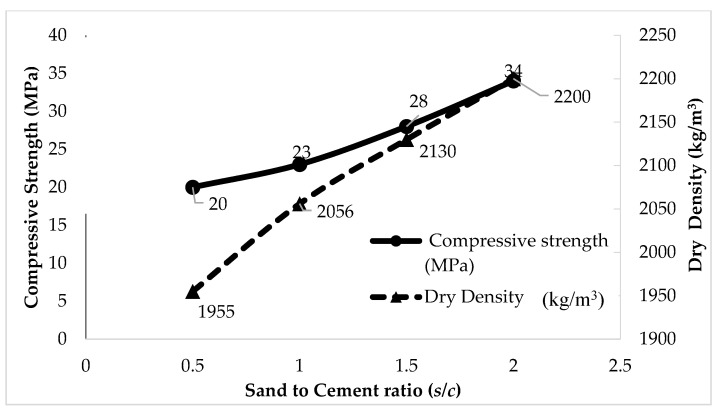
Relationship between compressive strength and density of mortar at four different *s/c* ratios.

Here, the relationship between compressive strength and dry density of mortar was studied. The target mortar density of 2100 kg/m^3^ has to remain with acceptable strength. The relationship between compressive strength and dry density, as well as the effect of sand cement ratio, are shown in Figure 3. Based on this relationship, it reveals that the compressive strength was directly proportional to the sand to cement ratio and density. The compressive strength of mortar increases with the increasing density due to increase in sand to cement ratio. It shows that the *s/c* = 2.0 mix achieved the highest compressive strength of 34 MPa as compared to others. In this study, optimal sand to cement ratio is chosen based on the target density of 2100 kg/m^3^. In most mixtures, *s/c* = 1.5 mixes show an acceptable density range of 2130 kg/m^3^ within the target density of 2100 kg/m^3^, which gives a compressive strength of 28 MPa.

### 4.2. Effect of Foam Dosage

#### 4.2.1. Effect of Foam Dosage on Workability

The fresh density, flow spread, number of drops, stability, and consistency of different percentages of foam dosage mixes were studied. The aim of this study is to obtain the desired density for the control mix 1600 kg/m^3^ in order to set a baseline to produce a control FC mix. Table 3 shows the workability of the control FC mixes at a different percentages of foam dosage. The flow spread value has a directly proportional relationship with the percentage of foam dosage. An increasing percentage of foam dosage causes an increase in the flow spread of fresh FC. Referring to the data, the flow spread value for foam dosages of 20%, 25%, 30%, and 35% was 200 mm, 205 mm, 208 mm, and 210 mm, respectively. It is observed that a higher percentage of foam dosage gives a higher workability of fresh FC. Even though the flow spread diameter gradually increases, the percentage of foam dosage also increasess and becomes more fluid, but the number of the drop of flow table decreases due to similar factors.

Furthermore, it is clear that when the percentage of the foam dosage increases, the design and fresh density of the control FC mixes decreases, with increasing foam dosage. This indicates that density and foam dosage have an inversely proportional relationship. The design density of FC at foam dosages of 20%, 25%, 30%, and 35% was 1934 kg/m^3^, 1875 kg/m^3^, 1817 kg/m^3^, and 1758 kg/m^3^, respectively. The fresh density of FC at foam dosages of 20%, 25%, 30%, and 35% was 1684 kg/m^3^, 1575 kg/m^3^, 1463 kg/m^3^, and 1355 kg/m^3^, respectively. There is a good relationship between design density and fresh density, whereby the fresh density of control FC mixes were decreased as higher percentages of foam dosage were added. This condition has a similar agreement with previous researchers by Bing et al. [8] and Wang and Tang [84], who reported that increasing the dosage of foam results in decreasing density of FC. Moreover, there is a good relationship between the stability and consistency of the control FC mixes’ density. The stability and consistency of the control FC mix at a different percentages of foam dosage are close to one.

#### 4.2.2. Effect of Foam Dosage on Densities and Compressive Strength

The dry density and 28-day compressive strength of the control FC specimens were investigated as shown in Table 4. The experimental results showed that although the fluidity of the control mixes was observed to gradually increase as the foam dosage increased, the 28-day compressive strength of control mixes rapidly decreases with increased foam dosage. It was shown that the compressive strength of 20% foam dosage was highest with 8.8 MPa, followed by 25% (7.5 MPa), 30% (4.2 MPa), and 35% (3 MPa). As compared to the control mortar, the compressive strength produced by the *s/c* = 1.5 mixes was 69% higher than the compressive strength of control FC at 28 days. It is concluded that compressive strength and foam dosage have an inversely proportional relationship. Furthermore, the dry density and fresh density of the control FC were way below that of the control mortar. Based on data, the dry density of 35% foam dosage was lowest with 1409 kg/m^3^, followed by 30% (1517 kg/m^3^), 25% (1625 kg/m^3^), and 20% (1734 kg/m^3^). However, the control FC shared the similar trend in mortar where the dry density was slightly higher than fresh density in ranges below 100 kg/m^3^. Additionally, this condition has a similar agreement with previous researchers Bing et al. [8] and Wang and Tang [84], found that increasing dosage of foam results in decreasing density and compressive strength of FC.

#### 4.2.3. Relationship between Compressive Strength and Density due to Foam Dosage

**Figure 4:**
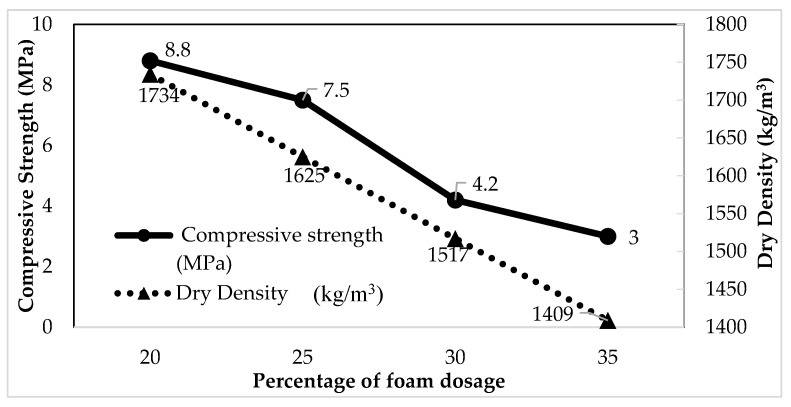
Relationship between compressive strength and density of control FC due to foam dosage.

Here, the relationship between compressive strength and density of FC was studied. The target control FC density of 1600 kg/m^3^ has to be maintained with acceptable strength. The relationship between compressive strength and density, as well as the effect of percentage of foam dosage, was shown in Figure 4. Based on this relationship, it was noticed that compressive strength was inversely proportional to the percentage of foam dosage and density. The 20% foam dosage mix achieved the highest compressive strength of 8.8 MPa as compared to others. From the graph, the higher compressive strength of control FC was owned by 20% of foam dosage but optimal foam dosage is chosen based on the target density of 1600 kg/m^3^. Thus, 25% of foam dosage was recommended due to its reasonable in density (1625 kg/m^3^), workability (205 mm), and 28-day compressive strength of the control FC (7.5 MPa).

### 4.3. Effect of Water Cement Ratio

#### 4.3.1. Effect of the Water–Cement Ratio on Workability

The fresh density, flow spread, number of the drop, stability, and consistency of different water–cement ratio mixes were studied. This study aims to obtain the desired density for control mix 1600 kg/m^3^ to set a baseline to produce a control FC mix. Table 3 shows the workability of control FC mixes at five different water–cement ratios. The flow spread value has a directly proportional relationship with water–cement ratio. Increasing water–cement ratio increases the flow spread of fresh FC. According to data, the maximum flow value was 215 mm on the 0.6 *w/c* mix and the minimum flow was 200 mm on the 0.4 *w/c* mix. Although the flow spread diameter increased and the water–cement ratio increased and became more fluid, the number of the drop of flow table decreased due to the same factor. According to Zhao et al. [42], the flow spread of FC containing GGBS as cement replacement increased gradually when the *w/c* ratio increased from 0.54 to 0.64. It shows that the amount of water affects the slurryness of FC, which achieves the target density. In addition, the flowability of fresh mix is related with the compressive strength of FC.

Additionally, it clearly shown that when the water–cement ratio increases, the fresh density of control FC mixes increase. This indicating that the fresh density and water–cement ratio has a directly proportional relationship. The fresh density of FC at water–cement ratio of 0.4, 0.45, 0.5, 0.55, and 0.6 was 1690 kg/m^3^, 1660 kg/m^3^, 1620 kg/m^3^, 1580 kg/m^3^, and 1530 kg/m^3^, respectively. This condition has a similar agreement with Risdanareni et al. [85] who reported that increasing the water–cement ratio results in slightly increasing the density of FC and then decreasing. In addition, there is a good relationship between the stability and consistency of control FC mixes density. The stability and consistency of control FC mix at different water–cement ratio are close to one. From the data, the consistency of the 0.5 *w/c* mix was equal to one and produced the density 1620 kg/m^3^ that was close to the desired density of 1600 kg/m^3^.

#### 4.3.2. Effect of the Water–Cement Ratio on Densities and Compressive Strength

The dry density and 28-day compressive strength of control FC specimens were studied, as shown in Table 4. According to data, the fluidity of control mixes was observed to gradually increase as the water–cement ratio increased, but the 28-day compressive strength of the FC slightly increased and then decreased with increasing water. It was shown that the highest compressive strength was 7.5 MPa with the *w/c* of 0.50 mix. This condition has a similar agreement with Liu, Zhao, Hu, and Tang [48] who reported that the compressive strength of FC increased first and then decreased when the water cement ratio increased. Other researchers, Zhao et al. [42] and Risdanareni et al. [85], reported that increasing the use of the water–cement ratio in FC lead to a reduction in compressive strength of FC. It can be concluded that compressive strength and water–cement ratio have an inversely proportional relationship.

#### 4.3.3. Relationship between Compressive Strength and Density due to the Water–Cement Ratio

**Figure 5:**
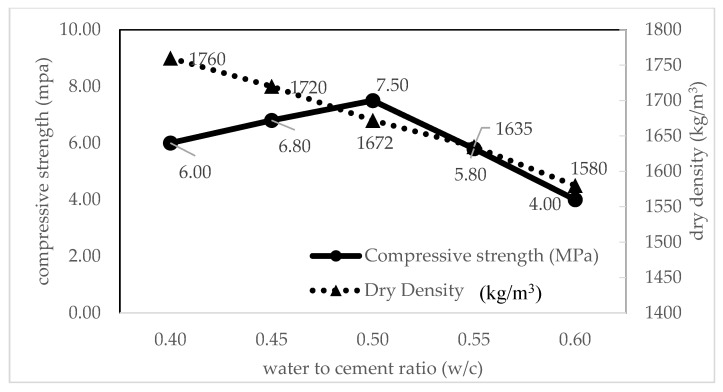
Relationship between compressive strength and density of control FC due to cement ratio.

Here, the relationship between compressive strength and density of foamed concrete was studied. The relationship between compressive strength and density, as well the effect of the water to cement (*w/c*) ratio is shown in Figure 5. Based on this relationship, it was noticeable that compressive strength was inversely proportional to the percentage of the *w/c* ratio and density. According to Mugahed et al. [43], water content of the mixes influence the compressive strength of FC. The increasing of the *w/c* ratio leads to a decrease in the compressive strength of FC [66]. As reported by Wee et al. [30], FC made with different *w/c* ratios produced different air void size, air void frequency, and spacing factor. Hence, air void size of FC increases with the increase in air content due to increasing the *w/c* ratio. Therefore, any variations in air void affect density, which significantly affects the compressive strength of FC. In this study, the highest compressive strength of FC was 7.5 MPa with the *w/c* of 0.50 mix as compared to others. Risdanareni et al. [85] also found that the compressive strength of FC with *w/c* of 0.50 was higher than FC with *w/c* of 0.4. In most mixtures, the 0.5 *w/c* mix shows a stable and consistent density range of 1620 to 1630 kg/m^3^ within the target density of 1600 kg/m^3^ that gives a compressive strength of 7.5 MPa.

### 4.4. Effect of Dilution Ratio

#### 4.4.1. Effect of Dilution Ratio on Workability

The fresh density, flow spread, number of the drop, stability, and consistency of five different dilution ratios mixes were studied. This study aims to obtain the desired density for the control mix 1600 kg/m^3^ to set a baseline to produce a control FC mix. Table 3 shows the workability of control FC mixes at different dilution ratios. The flow spread value have a directly proportional relationship with dilution ratios. Increasing dilution ratios will increase the flow spread of fresh foamed concrete. Based on data, the flow spread value at five different dilution ratios of 1:20, 1:25, 1:30, 1:35, and 1:40 was 215 mm, 225 mm, 230 mm, 235 mm, and 240 mm, respectively. From the experimental data, a higher dilution ratio of foaming agent gives higher workability of fresh foamed concrete. In addition, the flow spread diameter gradually increases and the dilution ratio increases and becomes more fluidity, but the number of the drop of flow table decreases due to the same factor. It is noted that when the dilution ratios increase, the design and fresh density of the control FC mixes decreases with an increase in the dilution ratio. This indicates that density and dilution ratio have an inversely proportional relationship.

The fresh density of FC at five different dilution ratios of 1:20, 1:25, 1:30, 1:35, and 1:40 was 1660 kg/m^3^, 1630 kg/m^3^, 1560 kg/m^3^, 1500 kg/m^3^, and 1480 kg/m^3^, respectively. There is a good relationship between design density and fresh density, where the fresh density of the control FC mixes decreased along, and equally, with their dilution ratio. This condition has a similar agreement with previous research which reported that increasing the dilution ratio results in decreasing the density of FC [8]. Kuzielova et.al [86] reported that lower concentrations of foaming agent increases the stability of the foam. The stability and consistency of the control FC mix at five different dilution ratios are close to one. From the data, the dilution ratio 1:25 give density 1608 kg/m^3^ that was close to the desired density of 1600 kg/m^3^.

#### 4.4.2. Effect of Dilution on Densities and Compressive Strength

The dry density and 28-day compressive strength of control FC specimens were studied as shown in Table 4. Although the fluidity of the control mixes was observed to gradually increase with the dilution ratios increasing, the average 28-day compressive strength of the control mixes rapidly decreases. It was shown that the compressive strength of 1:20 and 1:25 mix was highest with 10.20 MPa and 10 MPa then followed by 1:30 (6.2 MPa), 1:35 (4.5 MPa), and 1:40 (3.3 MPa). As compared to the control mortar, the compressive strength produces by *s/c* = 1.5 mixes was 63% higher than the compressive strength of control FC at 28 days. It can be concluded that the compressive strength and dilution ratios have an inversely proportional relationship. From experimental data, control FC shared a similar trend in mortar where the dry density was slightly higher than the fresh density in ranges below 100 kg/m^3^. The dry density of 1:40 mix was lowest with 1430 kg/m^3^ and followed by 1:35 (1460 kg/m^3^), 1:30 (1540 kg/m^3^), 1:25 (1608 kg/m^3^, and 1:20 (1640 kg/m^3^). Additionally, this condition has a similar agreement with previous research which found that increasing the dilution ratio results in the decreasing of density and the compressive strength of foamed concrete [76].

#### 4.4.3. Relationship between Compressive Strength and Density due to Dilution Ratio

**Figure 6:**
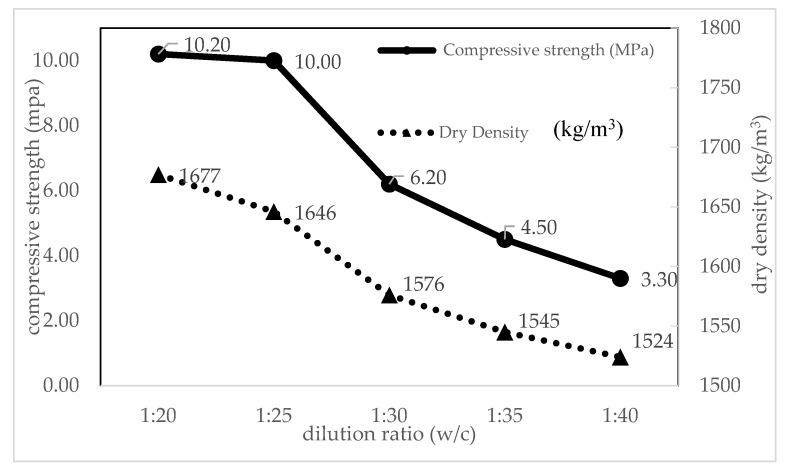
Relationship between compressive strength and density of control FC due to dilution ratio.

At this point, the relationship between compressive strength and density of foamed concrete is studied. The relationship between compressive strength and density, as well as the effect of dilution is shown in Figure 6. Based on the relationship, the compressive strength of control FC decreases with the increasing of the dilution ratio. It reveals that compressive strength was inversely proportional to the dilution ratios and density. The 1:20 and 1:25 mix achieved the highest compressive strength of 10.20 to 10.00 MPa as compared to others. The results showed that the optimal dilution ratio is 1:25 where compressive strength achieved the highest value 10.00 MPa with density being 1608 kg/m^3^.

### 4.5. Effect of PSBE Content

#### 4.5.1. Effect of Different Percentage of PSBE on Workability

The fresh density, flow table spread, number of the drop, stability, and consistency of different percentage of PSBE mixes were studied. This study aims to obtain the best mix proportions of FC containing PSBE (PFC). Table 3 shows the workability of control FC and five different percentages of PSBE (PFC 1 to PFC5). The flow spread value has an inversely proportional relationship with portions of PSBE. Increasing PSBE content decreases the flow spread of fresh foamed concrete. The results show 10%, 20%, 30%, 40%, and 50% of PSBE mixture have 215 mm, 205 mm, 200 mm, 150 mm, and 130 mm flow diameter, respectively. The reduction in the workability of the mix when PSBE added is probably due to increased particle surface of fine PSBE compared to cement. According to Mirza et al. [87], the workability of concrete mixtures will change due to the small particle size and the relatively higher surface area of pozzolan particles. Similarly, previous researchers Ali et al. [88] and Ahmad et al. [89] reported that concrete made with pozzolan materials has less workability than a control specimen produced with 100% cement. The stability and consistency of five PFC mixes were excellent, and the FC mix was equal to one.

#### 4.5.2. Effect of Different Percentage of PSBE on Densities and Compressive Strength

The dry density and 28-day compressive strength of control FC and five different percentages of PSBE (PFC 1 to PFC5) were studied as shown in Table 4. It was observed that the density of the control FC mix was slightly higher than the density of mixes with PSBE. Based on data, the lowest dry density was 1610 kg/m^3^, produced by PFC5 mix, and followed by 1620 kg/m^3^ (PFC4), 1641 kg/m^3^ (PFC3), 1632 kg/m^3^ (PFC2), and 1630 kg/m^3^ (PFC1). It was a similar trend with control FC mix where the dry density was slightly higher than fresh density in the range of below 100 kg/m^3^. In this study, the higher the PSBE content in the mixture, the lower is the density due to the specific gravity of the cement, which is more than PSBE. According to Nambiar and Ramamurthy [75], the density of FC depends on the filler type, such as fly ash, which has low specific gravity compared to fine sand. Moreover, the lowest density value in concrete incorporating finer rice husk ash is also due to the low specific gravity of RHA [90]. Other than that, mortar made with pozzolan materials, which have a lower density than the control specimen produced of 100% of cement, has a similar agreement with an available study on pozzolan materials [89]. The average compressive strength of FC containing PSBE was higher than the control FC. The compressive strength increased when PSBE is used as partial cement replacement. Among the PFC mixtures, PFC3 (30% PSBE) mix performed the highest compressive strength. The laboratory data show that although the density of PFC mixes was observed to gradually decrease with the PSBE percentage increasing, the average 28-day compressive strength of the PFC mixes slightly increased and then decreased when the PSBE content was increasing. It was shown that the compressive strength of PFC3 (30% PSBE) was highest with 23 MPa. As compared to the control FC, the compressive strength produced by PFC3 was 57% higher than the compressive strength of control FC at 28 days. In addition, compressive strength of PFC mixes exceeded the requirement for both non-loadbearing concrete masonry ASTM C129-17 [91] and load-bearing concrete masonry ASTM C90-16 [92] at 4.1 MPa and 13.1 MPa, respectively.

The presence of PSBE in the foamed concrete is the principal reason for the compressive strength of PFC higher than FC. It contains silica (SiO_2_) and alumina (Al_2_O_3_) in an active form which reacts with the calcium hydroxide (CH) that was produced during hydration of cement to generate more calcium silicate hydrate (CSH) gel in the presence of water. Pozzolanic reaction contributes to more CH being removed from the paste by reaction with PSBE depending on the curing condition, particularly at the early stage of curing. Furthermore, the reduction of voids in PFC is linked to an increase in strength that is evident from the pozzolanic effect by which more CSH produces denser specimens since FC is a porous structure. A similar influence of pozzolan material on the strength of foamed concrete was reported by [7,8,29,93,94,95].

#### 4.5.3. Relationship between Compressive Strength and Density due to Different Percentage of PSBE

**Figure 7:**
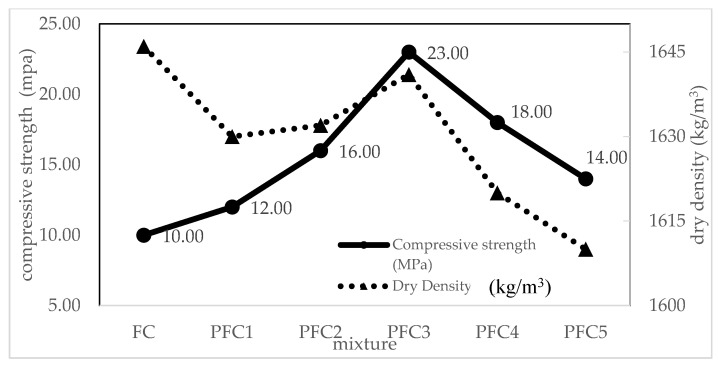
Relationship between compressive strength and density of PFC.

Currently, the relationship between compressive strength and density of FC was studied. The relationship between compressive strength and density, as well as the effect of PSBE content is shown in Figure 7. Based on the relationship, the compressive strength of PFC increases with the increasing of the PSBE content ratio. It revealed that the compressive strength of PFC was directly proportional to the PSBE content and density. The PFC3 mix achieved the highest compressive strength of 23 MPa with 1641 kg/m^3^ as compared to others.

In addition, the values of the regression analysis and the coefficient for each variable are shown in Table 5. The dependent variables (y) were workability, density, and compressive strength, while the independent variables (x) were sand to cement ratio, foam dosage, water to cement ratio, foaming agent dilution, and PSBE content. Based on the regression analysis, the results show strong correlation between the selected independent variables and dependent variables due to the R^2^ value closer to one. It indicates that the linear relationship is observed between selected independent variables with compressive strength and density. Nevertheless, the relationship between the water–cement ratio and compressive strength is curvilinear. The relationships were represented in the following equation.

(5) y= mx+c

## 5. Recommendations

The incorporation of PSBE significantly influences the density and compressive strength of foamed concrete. The results indicate that the compressive strength of FC depends on a density. The compressive strength of foamed concrete increase with increasing PSBE content up to 30% PSBE. However, the increment of PSBE percentage increases the water demand for the mix to maintain the same consistency. For future work, it is recommended that an investigation on the effect of PSBE combined with other pozzolan ash and chemical admixture on the properties of foamed concrete.

## 6. Conclusions

Findings of this study reveal the relationship between density and compression of foamed concrete developed with various ratio of sand cement, foam dosage, water to cement, dilution ratio, and PSBE content. According to the results, there is a directly proportional relationship between the ratio of sand–cement and PSBE content with density and compressive strength except for foam dosage, and water to cement and dilution ratio. It has been demonstrated that when the sand to cement ratio increases, the density and compressive strength of mortar increases with the increasing of the sand content. Consistently, there is a directly proportional relationship between dry density and the compressive strength of mortar. It indicates that the compressive strength of mortar increases with the increasing of density. In this study, optimal sand to cement ratio is chosen based on the target density of 2100 kg/m3. The use of 1:1.5 (cement: sand) ratio for mortar mix specified the best performance for density, workability, and 28-day compressive strength.

For the production of the control FC, there is an inversely proportional relationship between the foam dosage, water to cement ratio, and dilution ratio with density and compressive strength. Referring to the results, the increase in the percentage of foam dosage decreases the density and compressive strength of FC. It was noticed that the compressive strength decreases when the density of FC decreases. In this study, the use of a 25% foam dosage achieved the desired density of 1625 kg/m^3^ with 205 mm workability and 28-day compressive strength of 7.5 MPa. Similarly, the increasing of water cement ratio led to a decrease in compressive strength of FC. It was reported that the compressive strength of FC increased first and then decreased when the water cement ratio increased because the air void size of FC increases with the increase in air content due to increasing *w/c* ratio. In most mixture, the 0.5 *w/c* mix produced consistent results with density being 1630 kg/m^3^, 204 mm workability, and compressive strength of 7.5 MPa. Additionally, this condition has a similar trend in the increasing dilution ratio resulting in the decreasing of density and compressive strength of FC. The results showed that the optimal dilution ratio is 1:25 (1 L of foaming agent:25 L water) where the desired density for control FC 1630 kg/m^3^ with compressive strength of 10 MPa are met.

The incorporation of PSBE significantly influences the density and compressive strength of foamed concrete. Based on the relationship, the compressive strength of FC increases with increasing PSBE content up to 30% PSBE. However, beyond that the foamed concrete experiences strength reduction. It was revealed that the optimal mix of 30% PSBE as replacement of cement achieved the highest compressive strength of 23 MPa with 1641 kg/m^3^ as compared to others. This finding fulfils the requirement for strength of structural bearing load, whose requirement is more than 17 MPa by ASTM C 330-17a [96]. On the other hand, foamed concrete containing 30% PSBE has the potential to be used in construction applications.

## Figures and Tables

**Table 1:** Chemical composition of the OPC and PSBE.

Oxides (%)		PSBE	OPC
Silicon oxide	SiO_2_	55.82	16.05
Aluminum oxide	Al_2_O_3_	13.48	3.67
Calcium oxide	CaO	6.6	62.28
Ferrous oxide	Fe_2_O_3_	8.24	3.41
Magnesium oxide	MgO	5.94	0.56
Sulfur trioxide	SO_3_	1.05	4.10
Total of SiO_2_ + Al_2_O_3_ + Fe_2_O_3_		77.54	-
Loss on Ignition		0.18	1.2
Surface Area (BET) m^2^/g		8.484	4.459
Specific gravity		2.44	3.1
Surface Area (BET) m^2^/g		8.484	4.459
Specific gravity		2.44	3.1

**Table 2:** Mix proportion and densities of foamed concrete.

Mix	Design Density (kg/m^3^)	Fresh Density (kg/m^3^)	Cement (kg/m^3^)	PSBE (kg/m^3^)	Sand (kg/m^3^)	Water (kg/m^3^)	Foam (kg/m^3^)
1(*s/c* 0.5)	1981	1862	990.3	-	495.1	495.1	-
2(*s/c* 1.0)	2088	1967	835.3	-	835.3	417.7	-
3(*s/c* 1.5)	2167	2047	722.4	-	1083.5	361.2	-
4(*s/c* 2.0)	2227	2110	636.3	-	1272.6	318.1	-
5(*s/c* 1.5)	1934	1684	577.9	-	866.8	288.9	200
6(*s/c* 1.5)	1875	1575	541.8	-	812.6	270.9	250
7(*s/c* 1.5)	1817	1463	505.6	-	758.5	252.8	300
8(*s/c* 1.5)	1758	1355	469.5	-	704.3	234.8	350
9(*w/c* 0.40)	1625	1530	541.8	-	812.6	216.7	250
10(*w/c* 0.45)	1625	1580	541.8	-	812.6	243.8	250
11(*w/c* 0.50)	1625	1620	541.8	-	812.6	270.9	250
12(*w/c* 0.55)	1625	1660	541.8	-	812.6	298.0	250
13(*w/c* 0.60)	1625	1690	541.8	-	812.6	325.1	250
14(1:20)	1625	1660	541.8	-	812.6	270.9	250
15(1:25)	1625	1630	541.8	-	812.6	270.9	250
16(1:30)	1625	1560	541.8	-	812.6	270.9	250
17(1:35)	1625	1500	541.8	-	812.6	270.9	250
18(1:40)	1625	1480	541.8	-	812.6	270.9	250
FC	1600	1630	535.9	-	803.8	270.9	250
PFC1	1600	1619	482.3	53.6	803.8	274	250
PFC2	1600	1588	428.7	107.2	803.8	276	250
PFC3	1600	1557	375.1	160.8	803.8	280	250
PFC4	1600	1526	321.5	214.4	803.8	284	250
PFC5	1600	1495	267.9	267.9	803.8	288	250

**Table 3:** Workability of mortar and foamed concrete mixtures.

Mix	Design Density (kg/m^3^)	Fresh Density (kg/m^3^)	Flow Table Spread (mm)	Number of Drop	Stability	Consistency
1(*s/c* 0.5)	1981	1862	190	20	1.0	0.94
2(*s/c* 1.0)	2088	1967	195	20	1.0	0.94
3(*s/c* 1.5)	2167	2047	210	15	1.0	0.95
4(*s/c* 2.0)	2227	2110	215	15	1.0	0.95
5(20%)	1934	1684	200	15	0.97	0.87
6(25%)	1875	1575	205	13	0.97	0.84
7(30%)	1817	1463	208	12	0.96	0.81
8(35%)	1758	1355	210	10	0.96	0.77
9(*w/c* 0.40)	1625	1690	200	15	0.96	1.04
10(*w/c* 0.45)	1625	1660	202	13	0.97	1.02
11(*w/c* 0.50)	1625	1620	204	12	0.97	1.00
12(*w/c* 0.55)	1625	1580	210	11	0.97	0.97
13(*w/c* 0.60)	1625	1530	215	10	0.97	0.94
14(1:20)	1625	1660	215	10	0.99	1.02
15(1:25)	1625	1630	225	8	0.99	1.00
16(1:30)	1625	1560	230	7	0.99	0.96
17(1:35)	1625	1500	235	7	0.97	0.92
18(1:40)	1625	1480	240	7	0.97	0.91
FC	1625	1630	225	8	0.99	1.01
PFC1	1625	1622	215	15	0.99	1.01
PFC2	1625	1610	205	14	0.99	1.01
PFC3	1625	1615	200	12	0.98	1.01
PFC4	1625	1595	150	10	0.98	0.99
PFC5	1625	1580	130	10	0.98	0.99

**Table 4:** Densities and compressive strength of mortar and foamed concrete.

Mix	Fresh Density (kg/m^3^)	Dry Density (kg/m^3^)	Compressive Strength (MPa)
1(*s/c* 0.5)	1862	1955	20
2(*s/c* 1.0)	1967	2056	23
3(*s/c* 1.5)	2047	2130	28
4(*s/c* 2.0)	2110	2200	34
5(20%)	1684	1734	8.8
6(25%)	1575	1625	7.5
7(30%)	1463	1517	4.2
8(35%)	1355	1409	3.0
9(*w/c* 0.40)	1690	1760	6.00
10(*w/c* 0.45)	1660	1720	6.80
11(*w/c* 0.50)	1620	1672	7.50
12(*w/c* 0.55)	1580	1635	5.80
13(*w/c* 0.60)	1530	1580	4.00
14(1:20)	1660	1677	10.20
15(1:25)	1630	1646	10.00
16(1:30)	1560	1576	6.20
17(1:35)	1500	1545	4.50
18(1:40)	1480	1524	3.30
FC	1630	1646	10
PFC1	1622	1630	12
PFC2	1610	1632	16
PFC3	1615	1641	23
PFC4	1595	1620	18
PFC5	1580	1610	14

**Table 5:** Regression analysis of factors affecting density and compressive strength of foamed concrete.

Independent Variable	Dependent Variable	Correlation	R^2^	Expression
*s/c* ratio	Workability	Linear	0.953	y=18x+180
	Dry density	Linear	0.992	y=161.8*x* + 1883
	Compressive strength	Linear	0.980	y=9.4x+14.5
	Workability	Linear	0.959	y=0.66x+187.6
foam dosage	Dry density	Linear	0.999	y=−21.66*x* + 2166.9
	Compressive strength	Linear	0.962	y=−0.414x+17.26
	Workability	Linear	0.945	y=76x+168.2
*w/c* ratio	Dry density	Linear	0.997	y=−890*x* + 2118.4
	Compressive strength	curvilinear	0.361	y=−10x+11.2
	Workability	Linear	0.973	y=−1.2x+193
dilution ratio	Dry density	Linear	0.977	y=−11.36*x* + 1876.4
	Compressive strength	Linear	0.938	y=−0.386x+18.42
	Workability	Linear	0.883	y=−191.8x+235.8
%PSBE	Dry density	Linear	0.902	y=−0.93*x* + 1632
	Compressive strength	Linear	0.936	y=43x+8.8

## Data Availability

The data presented in this study are available on request from the corresponding author.
